# Systematic review of circulating MICRORNAS as biomarkers of cervical carcinogenesis

**DOI:** 10.1186/s12885-022-09936-z

**Published:** 2022-08-06

**Authors:** Neila Pierote Gaspar Nascimento, Thais Borges Gally, Grasiely Faccin Borges, Luciene Cristina Gastalho Campos, Carla Martins Kaneto

**Affiliations:** 1 Department of Health Sciences, Universidade Estadual de Santa Cruz, llhéus, BA Brazil; 2 Public Policies and Social Technologies Center, Federal University of Southern Bahia, Itabuna, BA Brazil; 3 Department of Biological Sciences, Universidade Estadual de Santa Cruz, Ilhéus, BA Brazil

**Keywords:** Circulating microRNA, microRNAs, Uterine cervical neoplasms, Cervical intraepithelial neoplasia, Biomarkers

## Abstract

**Background:**

Cervical cancer is a preventable disease, but it is a major public health problem despite having a good prognosis when diagnosed early. Although the Pap smear has led to huge drops in rates of cervical cancer and death from the disease, it has some limitations, making new approaches necessary for early diagnosis and biomarkers discovery. MiRNAs have been considered a new class of non-invasive biomarkers and may have great clinical value for screening early-stage cervical intraepithelial neoplasia. Well-designed studies have emerged as a necessary strategy for the identification of miRNAs that could be used safely and reliably for a differential diagnosis. This review aims to provide an up-to-date perspective on the assessment of circulating miRNA expression from precursor lesions to cervical cancer, identifying circulating miRNAs or specific miRNA signatures that can be used as potential biomarkers of different stages of cervical carcinogenesis.

**Methods:**

A systematic review was performed and searches were conducted in the PubMed, LILACS, and Scopus electronic databases.

**Results:**

Most studies involved Chinese ethnic women and searched for circulating miRNAs in serum samples. Thirty three microRNAs were evaluated in the eligible studies and 17 (miR-196a, miR-16-2, miR-497, miR-1290, miR-425-5p, hsa-miR- 92a, miR-1266, miR-9, miR-192, miR-205, miR-21, miR-152, miR-15b, miR-34a, miR-218, miR-199a-5p and miR-155-5p) showed up-regulation in women with precursor lesion and cervical cancer and 16 microRNAs showed decreased expression in these same groups of women compared to healthy controls (miR-195, miR-2861, miR-145, miR-214, miR-34a, miR-200a, let-7d-3p, miR-30d-5p, miR-638, miR-203a-3p, miR-1914-5p, miR-521, miR-125b, miR-370, miR-218 and miR-100).

**Conclusion:**

Therefore, defining promising circulating miRNAs or specific miRNA signatures of biological fluid samples can be useful for the screening, diagnosis, prognosis and clinical monitoring of women undergoing cervical carcinogenesis, but greater standardization of studies seems to be necessary for greater consolidation of information.

## Introduction

Cervical cancer (CC) is one of the most important public health problems worldwide, due to its high rates of morbidity and mortality [1], especially in less developed countries. Every year, approximately 570,000 women are diagnosed with CC in the world with an estimated 311,000 deaths, being considered the fourth most common type of cancer among women in the world, affecting relatively young women, between 30 to 50 years old [2]. Its development is often slow and it may take five to 15 years for its total progression from a precursor lesion, also called cervical intraepithelial neoplasia (CIN). When associated cellular alterations are not identified and/or treated early, they can develop into a localized tumor, called carcinoma in situ. This, in turn, can progress to invasive carcinoma, the most severe form, which can also become metastatic [3]. The staging of CC is determined by its dissemination and, according to the International Federation of Gynecology and Obstetrics (FIGO), as it increases, the estimated five-year survival for women affected by this neoplasm decreases.

Human Papillomavirus (HPV) infection, present in approximately 90% of cases [3], is considered the main risk factor for the development of CC. Although necessary, HPV infection alone is not enough to develop a cancerous disease and behavioral factors, both host and the virus-related, are also correlated [4]. According to their oncogenic potential, HPV types are classified as high or low oncogenic risk viruses [5] whose risk is directly related to the behavior of the viral genome in the host cell nucleus [6] [7].

Although Pap smear is the most used preventive strategy, this test also has its limitations [8], therefore, a better understanding of molecular mechanisms and genetic alterations associated with the pathogenesis of CC would be helpful to control this disease. In this context, non-invasive, specific, and sensitive biomarkers for early detection of CC are needed to reduce its morbidity and mortality worldwide [9].

Circulating microRNAs are considered a new class of these biomarkers due to their stability and presence in almost all body fluids [8]. They are also described as having great clinical value for screening early-stage different types of cancer, including CC. Well-designed studies, which aim to identify new biomarkers related to cervical carcinogenesis, emerged as a necessary strategy for the identification of miRNAs that could be used safely and reliably for a differential diagnosis. Different studies evaluating the expression of circulating microRNAs in CC show considerable divergence in the methodology used and also in the results obtained. Taking into account the great diversity and amount of information, as well as the divergences observed in these studies, the development of a systematic review on the gene expression of circulating microRNAs in cervical carcinogenesis would be important for the systematization and compilation of information available in the literature. Therefore, this review aimed to identify circulating miRNAs or specific miRNA signatures that could be used as potential biomarkers of different stages of cervical carcinogenesis, as well as provide an updated perspective on the evaluation of circulating miRNA expression from CC precursor lesions.

## Material and methods

As a study designed as a systematic literature review, the recommendations of the Preferred Reporting Items for Systematic Reviews and Meta-Analysis - PRISMA checklist were adopted [10]. The protocol for this review was submitted for registration in the International Prospective Register of Systematic Reviews (PROSPERO), under registration CRD42021259736. Permission from the ethics committee or institutional review board is not required for conducting a systematic review and meta-analysis.

### Study inclusion criteria

Inclusion criteria were: original studies; articles that evaluated the expression of one or more circulating microRNAs (serum, plasma or urine); published in English, from January 2011 to April 2022.

### Study exclusion criteria

**Fig. 1:**
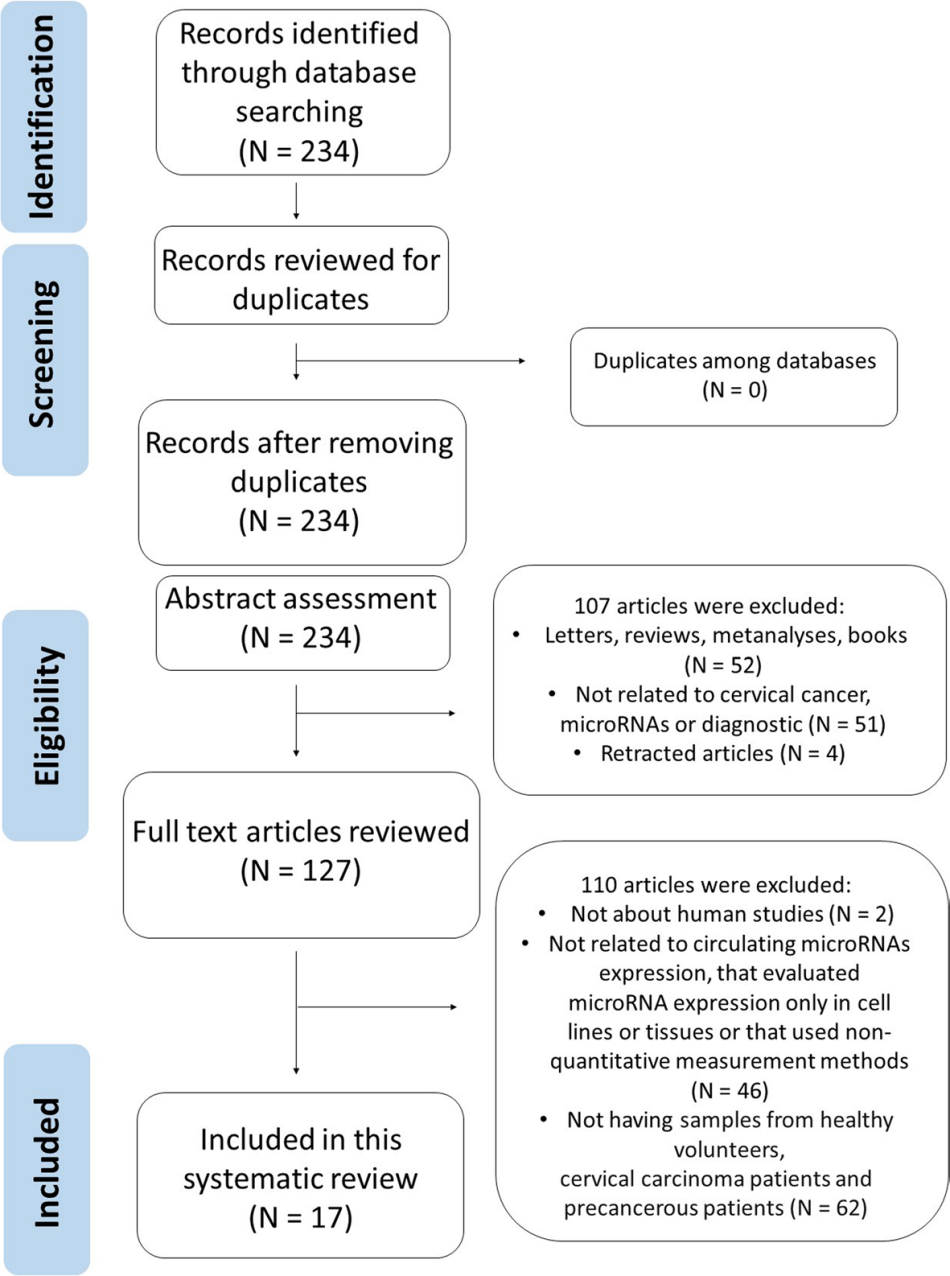
PRISMA flow chart

The articles were excluded for the reasons described in Fig. 1: articles that evaluated the expression of microRNAs only in tissue samples and cervical cells; studies unrelated to microRNA expression, cervical cancer or diagnosis; all those that did not assess the expression of circulating microRNAs; studies not performed in humans; research not carried out in three different sample groups (healthy control, cervical intraepithelial neoplasia and cervical cancer); studies that have evaluated the expression of microRNAs using qualitative or non-quantitative methodologies; conference abstracts; comments; literature reviews; letters to the editor; editorials; meta-analyses; clinical case reports; theses, dissertations, book chapters; studies with duplication in the databases and retracted articles.

### Searching and screening of literature

Pubmed (through pubmed.gov), LILACS (Latin American and Caribbean Health Sciences Literature – through https://lilacs.bvsalud.org/), and Scopus (through scopus.periodicos.capes.gov.br) databases were searched until April 2022. The search descriptors (terms) were as follows: cervical cancer OR uterine cervical neoplasms OR cervical intraepithelial neoplasia OR uterine cervical dysplasia AND circulating microRNAs OR plasma microRNAs OR serum microRNAs OR blood microRNAs OR cell-free microRNAs OR exosome microRNAs OR extracellular vesicles microRNAs.

### Data extraction

Eligible studies were independently and separately reviewed by two reviewers (N.P.G.N. and T.B.G.). Disagreements were resolved by discussions and mutual consensus. The necessary information and data related to the study design were extracted from each of the included studies as follows: authorship, year of publication, country of origin, type of study, subject’s ethnicity, number of sample groups, control group, CIN group, and cancer group data, in addition, miRNAs expression test methods and other variables described in Tables 1, 2 and 3. All information was tabulated by the reviewers in a spreadsheet (Microsoft Excel). Disagreements were resolved through discussion until an agreement was reached among reviewers.

**Table 1:** Characteristics of the samples in the included studies

	CONTROL GROUP (C)								CIN GROUP (CIN)			
Author	Year	Country	N	Mean Age (y)	HPV tested	HPV detection method	HPV infection	HPV type	N	Mean Age (y)	HPV tested	HPV detection method
Liu; Xin; Ma [11]	2015	China	50	N/I	No	N/A	N/A	N/A	T: 86 CIN I: 19 CIN II: 25 CIN III: 42	N/I	N/I	N/I
Zhang et al [8]	2015	China	193	N/I	N/I	N/I	N/I	N/I	186	N/I	N/I	N/I
Nagamitsu et al [12]	2016	Japan	31	39	N/I	N/I	N/I	N/I	T: 55 CIN I: 15 CIN II: 16 CIN III: 24	CIN I: 34 CIN II: 35 CIN III: 34	N/I	N/I
Sun et al [13]	2016	China	32	N/I	N/I	N/I	N/I	N/I	13	N/I	N/I	N/I
Kong et al [14]	2017	China	40	39	Yes	Liquid-Based Cytology	N/I	N/I	T: 68 CIN I: 21 CIN II: 23 CIN III: 24	39	Yes	Liquid-Based Cytology
Wei; Wen-Ming; Jun-Bo [15]	2017	China	120	N/I	N/I	N/I	N/I	N/I	120	N/I	N/I	N/I
Wang et al [16]	2018	China	50	N/I	N/I	N/I	N/I	N/I	HSIL: 50 LSIL: 50	N/I	N/I	N/I
Farzanehpour et al [17]	2019	Iran	36	36	Yes	PCR	Yes	16 66 68	18	47	Yes	PCR
Wang et al [18]	2019	China	42	40.94	Yes	N/I	No	N/A	T: 144 CIN I: 19 CIN II: 54 CIN III: 71	CIN I: 41.93 CIN II: 42.37 CIN III: 40.77	N/I	N/I
Yang; Zhang [19]	2019	China	50	43	Yes	N/I	No	N/A	T: 50 CIN I: 15 CIN II: 15 CIN III: 20	CIN I: 38.0 CIN II: 35.3 CIN III: 36.2	Yes	N/I
Zheng et al [20]	2019	China	50	50	N/I	N/I	N/I	N/I	T: 90 CIN I: 34 CIN II-III: 56	50	N/I	N/I
Zheng et al [21]	2020	China	41	42.71	N/I	N/I	N/I	N/I	40	43.88	N/I	N/I
Qiu et al [22]	2020	China	90	> = 50: 41 < 50: 49	N/I	N/I	N/I	N/I	45	> = 50: 20 < 50: 25	N/I	N/I
Aftab et al [23]	2021	Índia	50	N/I	Yes	PCR	Yes	16: 2	LSIL: 17 HSIL: 33	N/I	Yes	PCR
Hoelzle et al [24]	2021	Brasil	12	27,5	Yes	PCR	No	N/A	LSIL: 14 HSIL: 11	LSIL: 32 HSIL: 39	Yes	PCR
Ocadiz-Delgado et al [25]	2021	México	23	38	Yes	PCR	No	N/A	17	34	Yes	PCR
Yamanaka et al [26]	2021	Japão	34	42	N/I	N/I	N/I	N/I	64	CIN I: 34 CIN II: 35 CIN III: 34	N/I	N/I

	CIN GROUP (CIN)		CERVICAL CANCER GROUP (CC)								
Author	HPV infection	HPV type	N	Mean Age (y)	Tumor stage	Metast	HPV tested	HPV detection method	HPV infection	HPV type	Specim.
Liu; Xin; Ma [11]	N/I	N/I	105	<= 50: 64 > 50: 41	FIGO I-II: 74 III-IV: 31	Yes: 37 No: 68	Yes	N/I	Yes: 92 No: 13	N/I	Serum
Zhang et al [8]	N/I	N/I	184	N/I	N/I	N/I	N/I	N/I	N/I	N/I	Serum
Nagamitsu et al [12]	N/I	N/I	45	49	Ia: 7 Ib: 16 IIa: 7 IIb: 10 III: 3 IV: 2	N/I	N/I	N/I	N/I	N/I	Serum
Sun et al [13]	N/I	N/I	40	<= 60: 22 > 60: 18	Stage I: 12 Stage II, III and IV: 28	Yes: 29 No: 11	Yes	N/I	Yes: 28 No: 12	N/I	Serum
Kong et al [14]	Yes (all)	N/I	18	39	FIGO Ia - IIa	N/I	Yes	Liquid- Based Cytology	Yes (all)	N/I	Serum
Wei; Wen-Ming; Jun-Bo [15]	N/I	N/I	120	< 50: 59 > = 50: 61	FIGO I-II: 77 III: 43	Yes: 47 No: 73	Yes	N/I	Yes: 81 No: 39	N/I	Plasma
Wang et al [16]	N/I	N/I	50	N/I	N/I	N/I	N/I	N/I	N/I	N/I	Serum
Farzanehpour et al [17]	Yes	16 53	18	61	N/I	N/I	Yes	PCR	Yes	16	Serum
Wang et al [18]	N/I	N/I	15	49.73	N/I	N/I	N/I	N/I	N/I	N/I	Plasma
Yang; Zhang [19]	No	N/A	50	48	Stage I: 15 Stage II: 15 Stages III – IV: 20	N/I	Yes	N/I	No	N/A	Total Blood
Zheng et al [20]	N/I	N/I	63	50	N/I	N/I	N/I	N/I	N/I	N/I	Exosomes
Zheng et al [21]	N/I	N/I	40	50.39	FIGO I B1– I B2: 26 II A1– II B2: 12 N/I: 2	Yes: 9 No: 25 N/I: 6	N/I	N/I	N/I	N/I	Serum
Qiu et al [22]	N/I	N/I	112	> = 50: 53 < 50: 59	I: 90 IIA:22	Yes: 20 No: 92	N/I	N/I	N/I	N/I	Serum
Aftab et al [23]	Yes	16 LSIL: 4 HSIL: 24	50	N/I	I: 10 II: 17 III: 18 IV: 5	N/I	Yes	PCR	Yes	HG 16: 37 CS 16: 40 18: 2	Urine
Hoelzle et al [24]	Yes	LSIL 33: 2 Outros: 3 HSIL 16: 3 31: 1 Outros: 3	13	47	N/I	N/I	Yes	PCR	Yes	16: 4 18: 2 33: 2 Outros: 4	Plasma
Ocadiz-Delgado et al [25]	Yes	16	23	50	N/I	N/I	Yes	PCR	Yes	16	Serum
Yamanaka et al [26]	N/I	N/I	46	49	FIGO IA: 4 IB: 21 IIA: 4 IIB: 10 III: 3 IV: 4	Yes: 19 No: 16 N/I: 11	N/I	N/I	N/I	N/I	Serum

HPV Human Papillomavirus, N/I Not Informed, N/A Not appliable, CIN Cervical intraepithelial neoplasia, FIGO International Federation of Gynecology and Obstetrics, HSIL High grade squamous intraepithelial lesion, LSIL Low-grade squamous intraepithelial lesion, PCR Polymerase Chain Reaction, T Total, HG Histopathological grading, CS Clinical stage.

**Table 2:** MicroRNA candidate selection methods are found in included articles

Author	Year	Type of Global MicroRNA Expression profiling	If yes, were samples pooled?	If samples were not pooled, how many samples per group were analyzed in large-scale analysis?	Were candidate microRNAs selected by analysis of public microRNAs datasets?	Were candidate microRNAs selected by literature review?
Liu; Xin; Ma	2015	N/A	N/A	N/A	No	Yes
Zhang et al	2015	MicroRNA PCR panel	No	C: 5 CC: 4	No	No
Nagamitsu et al	2016	Microarray	No	C: 5 CC: 5	No	No
Sun et al	2016	N/A	N/A	N/A	No	Yes
Kong et al	2017	N/A	N/A	N/A	No	Yes
Wei; Wen-Ming; Jun-Bo	2017	N/A	N/A	N/A	No	Yes
Wang et al	2018	N/A	N/A	N/A	No	Yes
Farzanehpour et al	2019	N/A	N/A	N/A	No	Yes
Wang et al	2019	N/A	N/A	N/A	No	Yes
Yang; Zhang	2019	N/A	N/A	N/A	No	Yes
Zheng et al	2019	Sequencing	No	C: 23 CIN I: 5 CIN II-III: 59 SCC: 21 ACC: 13	No	No
Zheng et al	2020	N/A	N/A	N/A	No	Yes
Qiu et al [22]	2020	N/A	N/A	N/A	Yes	No
Aftab et al [23]	2021	N/A	N/A	N/A	Yes	No
Hoelzle et al [24]	2021	N/A	N/A	N/A	Yes	No
Ocadiz-Delgado et al [25]	2021	N/A	N/A	N/A	No	Yes
Yamanaka et al [26]	2021	N/A	N/A	N/A	No	Yes

*N/A* (Not applicable), *PCR* (Polymerase Chain Reaction), *C* (Control Group), *CC* (Cervical Cancer Group), *CIN* (Cervical Intraepithelial Neoplasia group), *SCC* (Squamous Cell carcinoma group), ACC (Adenocarcinoma group).

**Table 3:** Characteristics of the 12 studies included in our review

Author	Year	Detection Method	Normalize.	Method for expression level calculation	Differentially expressed microRNAs between HPV+ e HPV - samples	Differentially expressed microRNAs between CIN or CC and C	Up or down regulation description only	AUC	SEN	SPE	Main results description
Liu; Xin; Ma [11]	2015	qPCR	RNU6B	2^−ΔΔCt^	MiR-196a expression levels are not correlated to HPV infection	miR-196a	Yes	N/A	N/A	N/A	CC Patients showed a significantly higher level of serum miR-196a than CIN patients *p* < 0.05) and controls (*p* < 0.01). CIN patients expressed higher MiR-196a levels than C volunteers (*p* < 0.05). 67% of CIN III patients showed significantly higher expression of miR-196 than CIN II(32%) and CIN I (21%) patients. CC Patients with higher miR-196a serum levels had a poorer overall survival rate.
Zhang et al. [8]	2015	qPCR	cel-miR-67	2^−ΔΔCt^	N/I	miR-497 miR-16-2 miR-195 miR-2861	No	4 miRs: CC vs. C: 0.849 CC vs. NIC: 0.829 NIC vs. C: 0,734	4 miRs: CC vs. C: 73,1% CC vs. NIC: 71,4% NIC vs. C: 62,6%	4 miRs: CC vs. C: 88,4% CC vs. NIC: 67,2% NIC vs. C: 88,9%	MIR-195 expression level was significantly decreased, while miR-16-2* and miR-497 were significantly increased in CC patients, compared to CIN and control groups. miR-2861 expression levels are significantly decreased in CIN and CC patients compared to the control group.
Nagamitsu et al. [12]	2016	qPCR	miR-16	2^−ΔΔCt^	N/I	miR-1290	No	CC vs. C: 0.7957	CC vs. C: 90.3%	CC vs. C: 62.2%	MiR-1290 expression level was significantly elevated in CC patients compared to CIN and control patients. MiR-1290 increased expression was also related to the CC stage.
Sun et al. [13]	2016	qPCR	Geometric mean of miR-16 miR-223 and miR-31	2^−ΔΔCt^	N/I	miR-425-5p	Yes	N/A	N/A	N/A	MiR-425-5p expression levels were significantly the higher in CC group compared to CIN (*p*< 0.0001) or control groups (*p*< 0.0001). Elevated levels of miR-425-5p were significantly correlated with TNM stage (*p* = 0.0003) and lymph node metastasis (*p* = 0.0037). High levels of serum miR-425have expression that has been correlated with poor overall survival.
Kong et al. [14]	2017	qPCR	Cel-39-3p	2^−ΔΔCt^	MiR-92a expression levels are not correlated to HPV infection	hsa-mir-92a	No	0,83	69.6%	80.4%	Serum miR-92a levels were significantly higher in CIN and CC patients than in healthy subjects (*p*< 0.01). MiR-92a expression was also significantly higher in patients with advanced-stage CC than in those with early-stage CC.
Wei; Wen-Ming; Jun-Bo [15]	2017	qPCR	RNU6B	2^−ΔΔCt^	Lower plasmatic miR-145 expression levels significantly associated to HPV infection (p 0,016)	miR-145	No	CC vs. C: 0,848 CC vs. CIN: 0,828	CC vs. C: 81,7% CC vs. CIN: 91,7%	CC vs. C: 63,3% CC vs. CIN: 54,2%	MiR-145 plasma levels were significantly lower in CC patients than in CIN patients and healthy controls (p < 0.01). CC patients who achiea ved complete response to radiotherapy had higher plasma miR-145 levels than incomplete responders (p 0.005).
Wang et al. [16]	2018	qPCR	U6	2^−ΔΔCt^	N/I	miR-1266	Yes	N/A	N/A	N/A	Serum miR-126upregulatedulated in CC patients also correlates with metastatic progression. Serum MiR-1266 expression was significantly higher in patients with LSIL, HSIL and CC compared to healthy controls (*p*< 0.05). CC patients with high miR-1266 expression had lower overall survival rates than patients with low miR-1266 expression.
Farzanehpour et al. [17]	2019	qPCR	U6	2^−ΔΔCt^	N/I	miR-9 miR-192 miR-205	No	CC vs. C miR-9: 0,99 miR-192: miR-205: 0,96 CIN vs. C miR-9: 0,90 miR-192: 0,98 miR-205: 0,75 CC vs. CIN miR-9: 0,85 miR-192: 0,82 miR-205: 0,75	CC vs. C miR-9: 100% miR-192: 100% miR-205: 88,2% CIN vs. C miR-9: 77,8% miR-192: 83,3% miR-205: 66,7% CC vs. CIN miR-9: 52,9% miR-192: 58,8% miR-205: 35,3%	CC vs. C miR-9: 94,4% miR-192: 94,4% miR-205: 88,9% CIN vs. C miR-9: 94,4% miR-192: 94,4% miR-205: 88,9% CC vs. CIN miR-9: 94,4% miR-192: 83,3% miR-205: 94,4%	Increased expression levels of miR-9, miR-192 and miR-205 were observed in the CC group compared to normal samples (p< 0.0001). MiR-9, miR-192, and miR-205 expression levels were significantly increased in the CIN group compared to the control group. MiR-205 expression showed to be increased in the CC group compared to the CIN group (*p*< 0.0001).
Wang et al. [18]	2019	qPCR	U6	2^−ΔΔCt^	N/I	miR-21 miR-214 miR-34a miR-200a	No	CIN I- vs. CIN II+ miR-21: 0,613 miR-34a: 0,508 miR-200a: 0,615 miR-214: 0,505	N/I	N/I	MiRNA-21 plasma expression was increased in patients with cervical lesions (including CIN I, CIN II, CI, II,I and CC patients). MiRNA-21,−34a, and -200a expression levels were decreased in patients with severe cervical lesions (CIN I, CIN II, CIN, III and CC). Differences between these miRNAs expression levels were significant when comparing controls and CIN II e III patients(p< 0.05).
Yang; Zhang [19]	2019	qPCR	U6	2^−ΔΔCt^	N/I	miR-152	No	CIN vs. C: 0.831 CC vs. C: 0.935	N/I	N/I	MiR-152 expression levels were increased in CIN patients compared to control and higher in CC patients. Furthermore, miR-152 expression level in peripheral blood was higher in patients with high-grade CIN compared to those with low-grade CIN. MiR-152 expression levels also increased with the CC stage and were associated with serum SCC-Ag levels. A miR-152 expression reduction was observed after treatment.
Zheng et al. [20]	2019	Digital PCR	miR-128-3p miR-129-5p miR-320 let-7i-5p	R_score	N/I	let-7d-3p miR-30d-5p	No	let-7d-3p: 0.822 miR-30d-5p: 0.79 Combined: 0.828	N/I	N/I	Let-7d-3p and miR-30d-5p expression levels were significantly decreased in CIN II + group when compared to CIN I – group.
Zheng et al. [21]	2020	qPCR	cel-miR-39	2^−ΔΔCt^	N/I	miR-638 miR-203a-3p miR-1914-5p miR-521	No	CC vs. CIN miR-638: 0,681 miR-203a-3p: 0,651 miR-1914-5p: 0,717 miR-521: 0,716 CC vs. C miR-638: 0,734 miR-203a-3p: 0,660 miR-1914-5p: 0,626 miR-521: 0,742	CC vs. CIN miR-638: 85% miR-203a-3p: 42,5% miR-1914-5p: 82,5% miR-521: 62,5% CC vs. C miR-638: 80% miR-203a-3p: 75% miR-1914-5p: 85% miR-521: 80%	CC vs. CIN miR-638: 46,15% miR-203a-3p: 82,05% miR-1914-5p: 61,54% miR-521: 76,92% CC vs. C miR-638: 60,98% miR-203a-3p: 53,66% miR-1914-5p: 43,90% miR-521: 65,85%	Lower expression levels of miR-638, miR-203a-3p, miR-1914-5p and and MiR-521 were observed in the group compared to CIN. Furthermore, miR-638, miR-203a-3,p and miR-521 expression levels were lower in CC patients compared to healthy controls.
Qiu et al [22]	2020	qPCR	cel-miR-39	2^−ΔΔCt^	N/I	miR-21 miR-125b miR-370	No	CC vs. C miR-21: 0,783 miR-125b: 0,642 miR-370: 0,822 CC vs. CIN miR-21: 0,689 miR-125b: 0,735 miR-370: 0,821 Combined CC vs. C: 0,912 CC vs. CIN: 0,897	N/I	N/I	Higher miR-21 serum expression was observed in CC than in CIN and healthy women. The expression of miR-125b and miR-370 was lower in CC women compared to healthy and CIN patients. Combined expression of the three miRNAs performed well to identify early-stage CC compared to CIN or healthy controls. High levels of miR-21 and low levels of miR-125b and miR-370 were associated with metastasis and recurrences.
Aftab et al [23]	2021	qPCR	snRNA U6	2^−ΔΔCt^	Lower miRNA-145-5p expression in samples LSIL/HSIL HPV 16 + and CC HPV 16+ samples.	miR-21-5p miR-199a-5p miR-155-5p miR-34a-5p miR-145-5p miR-218-5p	No	miR-21-5p: 0,97 miR-199a-5p: 0,831 miR-155-5p: 0,719 miR-34a-5p: 0,888 miR-145-5p: 0,575 miR-218-5p: 0,609 Combined: 0,942 miR-21-5p miR-199a-5p miR-155-5p Combined: 0,969 miR-34a-5p miR-145-5p miR-218-5p Combined: 1 miR-21-5p miR-199a-5p miR-155-5p miR-34a-5p miR-145-5p miR-218-5p	miR-21-5p: 88 miR-199a-5p: 86.7 miR-155-5p: 93.8 miR-34a-5p: 89.1 miR-145-5p: 67.7 miR-218-5p: 83.3 Combined: 100 miR-21-5p miR-199a-5p miR-155-5p Combined: 100 miR-34a-5p miR-145-5p miR-218-5p Combined: 100 miR-21-5p miR-199a-5p miR-155-5p miR-34a-5p miR-145-5p miR-218-5p	miR-21-5p: 98 miR-199a-5p: 91.7 miR-155-5p: 88.3 miR-34a-5p: 91.7 miR-145-5p: 60 miR-218-5p: 72.7 Combined: 78.9 miR-21-5p miR-199a-5p miR-155-5p Combined: 92.8 miR-34a-5p miR-145-5p miR-218-5p Combined: 100 miR-21-5p miR-199a-5p miR-155-5p miR-34a-5p miR-145-5p miR-218-5p	Significant up-regulation of miR-21-5p, miR-199a-5p and miR-155-5p and down-regulation of miR-145-5p, miR-34a-5p and miR-218-5p was observed in women with LSIL/HSIL and CC when compared to healthy controls.
Hoelzle et al [24]	2021	qPCR	miR-23a-3p	2^−ΔΔCt^	Higher plasmatic miR-let-7a expression levels awithociated with HPV infection	miR-21 miR-let-7a miR-214	Yes	N/I	N/I	N/I	No significant differences were found between groups.
Ocadiz-Delgado et al [25]	2021	qPCR	Beta2mHu	2^−ΔΔCt^	N/I	miR-15b miR-34a miR-218	No	LSIL miR-34a: 0,9952 miR-15b: 0,6765 miR-218: 0,9872 CC miR-34a: 0,9505 miR-15b: 0,8173 miR-218: 0,8421	N/I	N/I	MiR-15b expression was higher in LSIL group and even higher in CC patients compared to healthy controls. MiR-34a and miR-218 expression increased in LSIL and decreased in CC, compared to control group.
Yamanaka et al [26]	2021	qPCR	miR-16	2^−ΔΔCt^	N/I	miR-100	No	0,879	91,2%	80,4%	MiR-100 serum expression was high in healthy women, intermediate in women with CIN and low in CC women.

*HPV* Human Papillomavirus, *C* Control Group, *CIN* Cervical Intraepithelial Neoplasia Group, *CC* Cervical Cancer Group, *AUC* Area Under Curve, *SEN* Sensibility, *SPE* Specificity, *qPCR* Quantitative Real-Time PCR, *N/I* Not Informed, *N/A* Not appliable.

### Quality assessment

To assess the risk of bias and applicability of the studies, the Quality Assessment of Diagnostic Accuracy Studies - QUADAS-2 tool was used. This scoring system combines four key domains: patient selection; index test; reference standard and flow and time. Each domain is assessed for risk of bias, such as high risk, unclear or low risk, and the first three domains are also assessed for applicability [27].

## Results

### Literature search results

Figure 1 shows that 234 articles were found in the first literature search according to the screening strategy. With no duplicates to remove, the 234 articles were submitted for the title and abstract assessment and 107 articles were excluded, because they had been published in the form of letters, reviews, editorials, case reports, expert opinions, protocols, conferences or meeting abstracts, comments or meta-analyses or were either related to cervical cancer, microRNAs or diagnostic or were retracted articles. The remaining 127 articles were submitted to full-text review, of which 110 articles were excluded because were not realized in human samples because they did not deal with circulating microRNAs, they evaluated the expression of microRNAs only in cell lines or tissues because they did not use quantitative measurement or not having samples from healthy volunteers, precancerous and cervical carcinoma patients. Seventeen studies met the criteria for the final analysis and were enrolled in this systematic review. These studies were finally assessed as described in the Preferred Reporting Items for Systematic Review and Meta-Analysis (PRISMA) guidelines for systematic review [10]. Tables 1, 2 and 3 show the characteristics of these studies.

### Characteristics of included studies

As shown in Table 1, of the 17 included articles, 11 were from China, 2 from Japan, 1 from Iran, 1 from India, 1 from Brazil and 1 from Mexico. First, we focused our analysis on the characterization of patients and samples, due to its important implications for the molecular diagnosis approach. The correct characterization of healthy donors, patients with cervical intraepithelial neoplasia and cervical cancer patients are necessary and correct information about clinical implications related to cervical cancer is imperative. The control group (C) included healthy women and a mean sample size of 56 patients (ranging from 12 to 193). The mean age was 39.81 years (ranging from 27.5 to 50 years), but six studies did not report the age of included donors [11] [8] [13] [15] [16] [23]. Still, in relation to the control group, seven studies reported that samples were tested for HPV infection [14] [17] [18] [19] [23] [24] [25], and one study declared that the samples were not tested [11] and other studies did not report any information on the assessment of HPV infection. Among the studies in which samples were tested for HPV infection, one reported using the liquid-cytology method [14], and four reports of HPV infection were evaluated by PCR [17] [23] [24] [25], identifying the presence of HPV types 16, 66 and 68. Two studies reported that HPV infection was not detected in women in the control group [18] [19], without mentioning the method used for HPV detection. A total of 1171 women were included in the cervical intraepithelial neoplasia (CIN) group in all included studies, with an average sample size of 69 patients (ranging from 13 to 186), but only 9 studies discriminated the number of affected women according to stage lesion. The mean age among studies that reported this information was 38.2 years (ranging from 32 to 50). Six studies reported that CIN samples were tested for HPV infection [14] [17] [19] [23] [24] [25] and the others did not report any information about HPV infection in this patient group. Kong et al. [14] reported HPV infection in all patients included in the CIN group and the same was reported by Farzanehpour et al. [17], who also reported that HPV types 16 and 53 were identified. Aftab et al. [23] and Ocadiz-Delgado et al. [25] reported that HPV type 16 was identified and Hoelzle et al. [24] reported the identification of HPV types 16, 31, and 33, among others. Yang and Zhang reported that none of the patients included in the CIN group had HPV infection [19]. The cervical cancer group (CC) included an average of 58 patients (ranging from 13 to 184) and the mean age among the studies that reported this information, was 49.31 years old (ranging from 39 to 61). Ten studies reported the tumor stage according to the International Federation of Gynecology and Obstetrics (FIGO) and 161 women were diagnosed with metastatic cervical cancer while 285 were not, but 11 studies did not report any information related to the presence of metastasis. Regarding the histological type, most studies diagnosed squamous cell carcinoma as the most prevalent: LIU; XIN; MA (carcinoma: 84, others: 21) [11], NAGAMITSU et al., (carcinoma: 38, adenocarcinoma: 06) [12], WEI; WEN-MING; JUN-BO (squamous cell carcinoma: 101, adenocarcinoma: 19) [15], YANG; ZHANG (epithelial cell carcinoma: 39, adenocarcinoma: 11) [19], ZHENG et al.(epithelial cell carcinoma) [21], QIU et al. (carcinoma: 107, adenocarcinoma: 05) [22], YAMANAKA et al. (carcinoma: 37, adenocarcinoma: 09) [26]. Squamous cell carcinoma was the prevalent type in all CC women in Aftab et al. [23] and Ocadiz-Delgado et al. [25] studies. Wang et al. [16] reported having found both histological types but did not discriminate the prevalence. Only Sun et al. [13] found a higher number of adenocarcinoma-type cases in relation to squamous cell carcinoma.

Nine studies reported that CC samples were tested for HPV infection and Kong et al. [14] found that all the women included had HPV infection, as well as patients in the study by Farzanehpour et al. [17] and Ocadiz-Delgado et al. [25] who also identified HPV type 16. In their study, Liu, Xin and Ma [11] reported that 92 patients had HPV infection and 13 did not. Sun et al. [13] found HPV-positive patients, while 12 had negative results. Wei, Wen, Mingand Jun-Bo [15] reported that 81 women had HPV infection and 39 did not. Yang and Zang reported that all patients included in the CC group did not have this type of infection [19].

Serum samples were the specimens evaluated in 11 articles, 3 studies analyzed plasma samples, 1 analyzed total blood, 1 evaluated microRNA expression in exosomes, and 1 analyzed urine. Three of the 17 included studies employed some type of high-throughput analysis for microRNA candidate selection, of which 1 used sequencing [20], 1 performed microarray analysis [12] and 1 employed some type of microRNA panel PCR [8]. Regarding the type of samples evaluated in these high-throughput analyses, all articles used non-pooled samples, 11 target miRNAs were selected from literature and 3 target microRNAs were selected by database analysis (Table 2).

Quantitative Real-time PCR was used to evaluate microRNAs expression in almost all studies, U6 and RNU6B microRNAs were the most used endogenous reference microRNAs for normalization purposes, but other microRNAs such as MiR-16, MiR-223, cel-miR-39 and cel-miR-67 were also found in the included studies, reinforcing the importance of to evaluate the choice of a normalization method to minimize quantization errors and technical variability in experiments (Table 3). Five studies reported assessment of microRNAs expression comparing patients with and without HPV infection. Liu, Xin, and Ma [11] verified that the expression levels of MiR-196a were not correlated with HPV infection and the same was observed by Kong et al. [14] for MiR-92a. Wei, Wen-Ming and Jun-Bo [15] found that low plasma miR-145 level was significantly associated with HPV infection. Aftab et al. [23] also observed reduced expression of miRNA-145-5p in LSIL/HSIL HPV 16+ and CC HPV 16+ samples. Hoelzle et al. [24] found high plasmatic levels miR-let-7a in HPV+ samples. The expression of 33 microRNAs was evaluated in the eligible studies included in this review and 17 microRNAs (miR-196a, miR-16-2, miR-497, miR-1290, miR − 425-5p, hsa-miR-92a, miR-1266, miR-9, miR-192, miR-205, miR-21, miR-152, miR-15b, miR-34a, miR-218, miR-199a-5p and miR-155-5p) were found to show expression differential in cervical carcinogenesis. Only miR-21 was differentially expressed in more than one study [18] [22] [23].

The AUC value was calculated in some studies with the aim of evaluating the diagnostic potential of microRNAs in cervical carcinogenesis. When comparing the CC group the controls, the AUC values were - miR-1290: 0.7957 [12]; miR-145: 0.848 [15] and miR-152: 0.935 [19]. The AUC value of 0.849 was obtained for miR-497, miR-16-2*, miR-195 and miR-2861 when combined to form a panel [8]. The AUC value for MiR-21, miR-125b and miR-370 was 0,912 [22]. Elevated AUC values were also observed for miR-9: 0.99; miR-192: 1 and miR-205: 0.96 [17], as well as for miR-638: 0.734; miR-203a-3p: 0.660; miR-1914-5p: 0.626 and miR-521: 0.742 [21] and miR-21: 0,783; miR-125b: 0,642 and miR-370: 0,822 [22]. When comparing patients in the CC and CIN groups, the following AUC values were obtained: miR-145: 0.828 [15]; miR-152: 0.831 [19]; miR-497, miR-16-2*, miR-195 and miR-2861 combined to form a panel: 0.829 [8]; miR-9: 0.85, miR-192: 0.82 and miR-205: 0.75 [17]; miR-638: 0.681, miR-203a-3p: 0.651, miR-1914-5p: 0.717 and miR-521: 0.716 [21]; miR-21: 0,689; miR-125b: 0,735 and miR-370: 0,821 [22]. The AUC value of 0.897 was obtained for miR-21, miR-125b e miR-370 when combined [22]. Comparison of CIN and healthy group resulted in AUC values: 0.831 for miR-152 [19]; 0.734 for combination of miR-497, miR-16-2*, miR-195 and miR-2861 [8]; 0.90, 0.98 and 0.75 for miR-9, miR-192 and miR-205, respectively [17]. The AUC value found was 0.83 for hsa-miR-92a [14], 0,879 for miR-100 [22], 0.822 and 0.79 for let-7d-3p and miR-30d-5p and 0.828 for the combination of both [20]. The AUC value was also calculated to differentiate between CC women who responded completely and incompletely to radiotherapy and a value of 0.801 was obtained for MiR-145 [15]. Comparisons between CIN- group (healthy control and CIN I cases) and CIN+ group (CIN II, CIN III and cervical cancer) were also performed, resulting in the following AUC values: miR -21: 0.613, miR-214: 0.505, miR-34a: 0.508 and miR-200a: 0.615 [18]. Ocadiz-Delgado found that AUC values were calculated for samples of patients with LSIL and the values found were: miR-34a: 0,9952, miR-15b: 0,6765 and miR-218: 0,9872, and for samples of women CC: miR-34a: 0,9505, miR-15b: 0,8173 and miR-218: 0,8421 [25]. Individual AUC values were found for miR-21-5p: 0,97, miR-199a-5p: 0,831, miR-155-5p: 0,719, miR-34a-5p: 0,888, miR-145-5p: 0,575, miR-218-5p: 0,609 [23].

In summary, 12 of 17 included studies described microRNAs with diagnostic potential for precursor lesion and cervical cancer with diagnostic performance ≥0.70. Among all 33 microRNAs evaluated, 25 showed diagnostic performance ≥0.70 (miR-497, miR-16-2, miR-195 and miR-2861 when combined to form a panel; miR-1290; miR-145; miR-9, miR-192 and miR-205; miR-152; miR-638, miR-1914-5p and miR-521; hsa-miR-92a; let-7d-3p and miR-30d-5p; miR-21, miR-125b and miR-370; miR-15b, miR-34a and miR-218; miR-100; miR-199a-5p and miR-155-5p).

Some microRNAs showed excellent diagnostic performance. The AUC values for miR-9, miR-192, and miR-205 were 0.99, 1, and 0.96 (when comparing the group of women with cervical cancer and healthy controls), and 0.90 and 0.98 (when comparing the group of CIN patients and the healthy controls) [17]. Excellent results were also obtained for miR-152 which showed an AUC value of 0.935 [19]. When combined to compare CC women and controls, miR-21, miR-125b and miR-370 showed an AUC value of 0,912 [22]. In LSIL women, AUC values of 0,9952 (miR-34a) and 0,9872 (miR-218) were found and in CC women an AUC value of 0,9505 for miR-34a was obtained [25]. Excellent performance was also observed for miR-21-5p (AUC: 0,97) and for combined miR-21-5p, miR-199a-5p and miR-155-5p (AUC: 0,942), and miR-34a-5p, miR-145-5p and miR-218-5p (AUC: 0,969) [23].

Among 8 studies that reported sensitivity, 5 exceeded 70% when comparing the CC and C groups. Among the 33 evaluated microRNAs, 19 showed sensitivity ≥70% (miR-497, miR-16-2, miR-195 and miR-2861 when combined to form a panel; miR-1290; miR-145; miR-9, miR-192 and miR-205; miR-638, miR-203a-3p, miR-1914-5p and miR-521; miR-100; miR-21-5p, miR-199a-5p, miR-155-5p, miR-34a-5p and miR-218-5p). When comparing the CIN and C groups, eight microRNAs showed sensitivity ≥80% (miR-1290, miR-145, miR-9, miR-192, miR-205, miR-638, miR-1914-5p and miR-521) and of these, miR-9 and miR-192 showed 100% sensitivity.

Eight studies reported specificity and, among them, 2 exceeded 80% when comparing the CC and C groups. Among the 33 evaluated microRNAs, 15 showed specificity ≥80% (miR-497, miR-16-2, miR-195 and miR-2861 when combined to form a panel; miR-9, miR-192, miR-205 and miR-92a; miR-100; miR-21-5p, miR-199a-5p, miR-155-5p and miR-34a-5p, when combined, miR-145-5p and miR-218-5p). MiR-9, miR-192 and miR-21-5p also had the highest specificity values (94.4 and 98%). Table 3 also shows the main results obtained for each one of the 17 eligible studies.

Thirty three differentially expressed microRNAs are shown in Table 4 that also shows that 17 microRNAs (miR-196a, miR-16-2, miR-497, miR −1290, miR-425-5p, hsa-miR-92a, miR-1266, miR-9, miR-192, miR-205, miR-21, miR-152, miR-15b, miR-34a, miR-218, miR-199a-5p and miR-155-5p) were up-regulated in CIN and CC patients compared to healthy controls and 16 microRNAs (miR-195, miR-2861, miR-145, miR-214, miR-34a, miR-200a, let- 7d-3p, miR-30d-5p, miR-638, miR-203a-3p, miR-1914-5p, miR-521, miR-125b, miR-370, miR-218 and miR-100) were downregulated in these same groups.

**Table 4:** Up and downregulated microRNAs expressed in patients with CIN and CC compared to healthy controls reported in the included studies

Upregulated miRNAs*	Downregulated miRNAs*
miR-196a [11]	miR-195 e miR-2861 [8]
miR-16-2* e miR-497 [8]	miR-145 [15]
miR-1290 [12]	miRNA-214, −34a e -200a [18]
miR-425-5p [13]	let-7d-3p e miR-30d-5p [20]
miR-92a [14]	miR-638, miR-203a-3p, miR-1914-5p e miR-521 [21]
miR-1266 [16]	miR-125b and miR-370 [22]
miR-9, miR-192 e miR-205 [17]	miR-34a and miR-218 [25]
miRNA-21 [18]	miR-100 [26]
miR-152 [19]	miR-34a-5p, miR-145-5p and miR-218-5p [23]
miR-21 [22]	
miR-15b, miR-34a and miR-218 [25]	
miR-21-5p, miR-199a-5p and miR-155-5p [23]	

* When comparing CIN and CC patients to healthy controls

### Methodological quality assessment

**Fig. 2:**
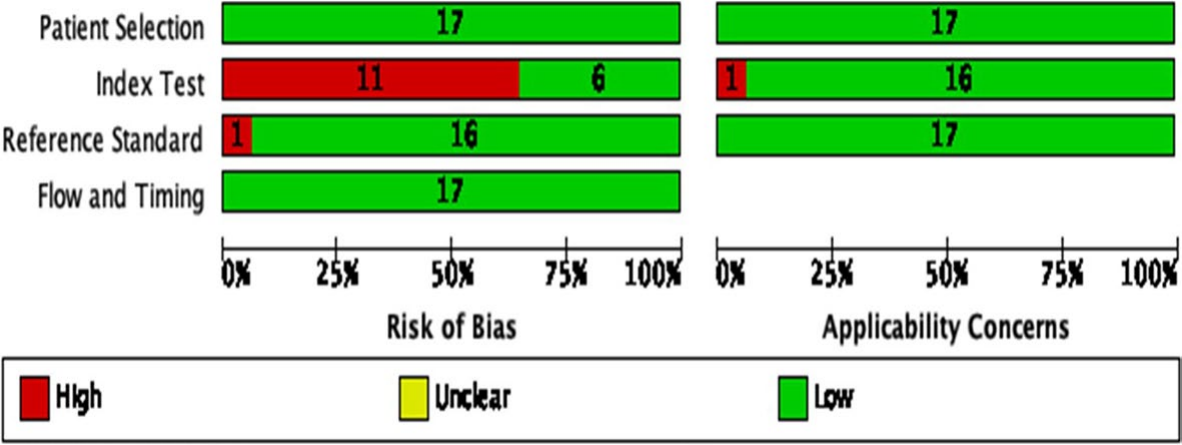
Quality assessment of studies using the Quality Assessment of Diagnostic Accuracy Studies 2 score system

QUADAS-2 was carried out for the 17 included studies for quality assessment. Quality assessment of included studies revealed a generally good quality. However, potential risk of bias was detected in our review and high-risk bias was found in eleven studies in the index test domain. The risk of bias and applicability concerns graph for included studies were presented in Fig. 2.

## Discussion

Despite having one of the highest potentials for prevention and cure among all neoplasms [28], cervical cancer is still considered a public health problem due to its high morbidity and mortality rates, affecting relatively young women, in the age group of 30 to 50 years old [2]. This type of cancer also has a good prognosis when diagnosed early and, although the Pap smear test is the most used preventive strategy for cervical cancer screening, this test also has its limitations [8], making new approaches necessary to achieve prevention, early diagnosis, treatment strategies, and prognosis to control this disease.

Due to their stability, circulating microRNAs, small non-coding RNAs that can be found freely in body fluids such as serum, plasma and urine, have been considered a new class of minimally invasive biomarkers and may have a great clinical value for early screening of cervical intraepithelial neoplasia stage. Studies aimed at the identification of new biomarkers related to cervical carcinogenesis emerged as a necessary strategy for the determination of microRNAs that could be used safely and reliably for a differential diagnosis.

In this review, we provided an updated perspective on the assessment of circulating miRNA expression from cervical cancer precursor lesions, identifying circulating miRNAs or specific miRNA signatures that can be used as potential biomarkers of different stages of cervical carcinogenesis. We identified 17 different studies, most involving women Chinese ethics, searching for circulating microRNAs differentially expressed in serum samples and using the literature review as a method for selecting microRNA candidates. However, the heterogeneity of the study was large. The sample size was found to be satisfactory (minimum of 50 patients per group) in the majority of included studies, but most of them failed to provide essential information about the characterization of CC, CIN and control evaluated samples. The description of the participant characteristics, such as age, type of lesion, tumor stage and presence of metastasis, whether the patients are undergoing chemotherapy are important to define whether the study findings can be generalized to show their possible limitations. The presence of HPV infection, for example, can also potentially alter the expression of circulating microRNAs [29] [30], which have been shown to play an important role in many physiological and pathological processes such as viral infections and oncogenesis. In this context, all samples included should must be tested for HPV infection in order to avoid the effect of this viral infection on microRNA expression, but only 6 of the eligible studies tested women from all samples group for HPV infection and some studies included women with and without HPV infection, which cause measurement errors.

Methodological differences in the same execution of experiments applied to the identification of differentially expressed circulating microRNAs were also found, potentially influencing the screening of circulating microRNAs for NIC and CC detection. The expression MicroRNAs and the final quantitative results, for example, can be greatly altered using different normalization methods [31] [32]. The absence of a universal quantitative evaluation for the expression of microRNAs and the consensus on the ideal endogenous reference microRNA to be used for the normalization of microRNA expression data from patients with NIC, CC and other types of cancer is another factor. Even though most used endogenous reference genes (RNU6B, cel-miR-39, U6 snRNA, and miR-16) were found in the included studies, other unusual microRNAs, such as MiR-128-3p, MiR-129-5p, let7i-5p, MiR-223 and MiR-31, were also found, reinforcing the importance of the development of a rigorous normalization strategy that avoids measurement errors.

The expression of 33 microRNAs was evaluated in the eligible studies included in this review and 17 of these microRNAs (miR-196a, miR-16-2, miR-497, miR-1290, miR-425-5p, hsa-miR- 92a, miR-1266, miR-9, miR-192, miR-205, miR-21, miR-152, miR-15b, miR-34a, miR-218, miR-199a-5p and miR-155-5p) were shown to be upregulated in women with precursor lesion and cervical cancer [8, 11–14, 16–19, 22, 25] and 16 microRNAs showed decreased expression in these same groups of women compared to healthy controls (miR-195, miR-2861, miR-145, miR-214, miR-34a, miR-200a, let-7d-3p, miR-30d-5p, miR-638, miR-203a-3p, miR-1914-5p, miR-521, miR-125b, miR-370, miR-218 and miR-100) [8, 15, 18, 20–23, 25, 26].

Only miR-21 was found to be differentially expressed in more than one study [18, 22, 23]. MiR-9 and miR-192 had the highest value of AUC, sensibility and specificity [17]. Therefore, defining promising circulating miRNAs or specific miRNA signatures from biological fluid samples can be useful for screening, diagnosis, prognosis and clinical follow-up of women undergoing cervical carcinogenesis, but greater standardization of studies seems to be necessary for greater consolidation of information to be obtained.

## Conclusion

In conclusion, this systematic review suggests that circulating microRNAs have great potential to be used in the diagnosis of CIN and CC, but future studies should consider a more rigorous standardization sample characterization especially in evaluating HPV infection. Differential expression of miR-21 and elevated AUC values, sensitivity and specificity observed for miR-9 and miR-192 suggest that these are promising candidates, although further studies with larger samples and women of different ethnical groups are necessary. Furthermore, these studies should also consider the combination of microRNA biomarkers already evaluated for CIN and CC detection, in order to enhance diagnostic power.

## Data Availability

Not applicable.

## Abbreviations

AUC: Area Under Curve
CC: Cervical cancer
CIN: Cervical Intraepithelial Neoplasia
FIGO: International Federation of Gynecology and Obstetrics
HPV: Human Papillomavirus
HSIL: High grade squamous intraepithelial lesion
LSIL: Low-grade squamous intraepithelial lesion
N/A: Not applicable
N/I: Not Informed
PCR: Polymerase Chain Reaction
PRISMA: Preferred Reporting Items for Systematic Reviews and Meta-Analysis
PROSPERO: International Prospective Register of Systematic Reviews
qPCR: Quantitative Real-Time PCR
QUADAS-2: Quality Assessment of Diagnostic Accuracy Studies
SEN: Sensibility
SPE: Specificity
